# Advancing passenger next-station prediction via collaborative knowledge graph representational learning

**DOI:** 10.1038/s41598-026-57645-5

**Published:** 2026-06-16

**Authors:** Xiaoqi Duan, Jianlong Wang, Zhibang Xu, Wenxin Teng, Youliang Tian

**Affiliations:** 1 State Key Laboratory of Public Big Data, College of Computer Science and Technology, Guizhou University, Guiyang, 550025 China; 2 School of Mathematies and Computer Science, Shaanxi University of Technology, Hanzhong, 723000 China; 3 State Key Laboratory of Regional and Urban Ecology, Institute of Urban Environment, Chinese Academy of Sciences, Xiamen, 361022 China; 4 Police Command College, China People’s Police University, Langfang, 065000 China

**Keywords:** Next-station prediction, Knowledge graph, Representational learning, Smart card data, Reinforcement learning, Population dynamics, Sustainability, Geophysics

## Abstract

Conventional passenger next station prediction models are often restricted by rigid graph structures, failing to account for the dynamic interactions between passengers and stations. This limitation results in an insufficient representation of travel patterns and associated knowledge. To address these shortcomings, this paper proposes a novel approach that integrates reinforcement learning with knowledge graphs to enable a holistic fusion of heterogeneous data. The proposed method enriches the environmental variables within reinforcement learning frameworks and introduces collaborative updating mechanisms for representing passenger-station interactions based on human travel knowledge graphs. These enhanced representations are then employed to improve the accuracy of next-station prediction. To evaluate the effectiveness of the approach, ablation experiments and comparative analyses with classical algorithms were conducted. The results demonstrate that the proposed method significantly outperforms existing models in predicting next-stations, routes, and travel distances, establishing its efficacy in capturing complex passenger travel behaviors.

## Introduction

In recent years, with the rapid advancement of location tracking technology, individuals have amassed substantial volumes of travel data in their daily routes, including trajectories, smart card transactions, and check-in records. These datasets not only capture passengers’ real-time locations but also encompass diverse features of human mobility, presenting unparalleled opportunities for uncovering spatiotemporal travel patterns^1–4^. Accurate next-station prediction is vital for addressing modern urban mobility challenges, including 1)reducing uncertainty in wait times and recommending congestion-free routes, 2) Enabling dynamic resource allocation (e.g., adjusting train frequencies based on predicted demand), and 3) Identifying travel pattern shifts for long-term infrastructure optimization. Nonetheless, the effective organization and integration of spatiotemporal data play a pivotal role in accurately predicting travel next-station^5,6^.

Human mobility prediction has been a focal point of extensive research. Early studies predominantly utilized Matrix Factorization and Markov Chains, which became mainstream methods for sequential prediction. However, Markov chain models, such as the one proposed by Rendle^7^, often fall short of achieving optimal results due to their reliance on a limited set of features and their failure to account for complex behavioral patterns. These models are particularly limited in capturing temporal dependencies, leading to suboptimal performance. In recent years, deep learning technologies have demonstrated remarkable dominance across various research domains. Recurrent Neural Networks (RNNs) and their variants have emerged as standard solutions for analyzing sequential data^8,9^. For example, LSTPM^10^ was proposed to address both long-term and short-term user preferences by designing a specialized network for long-term preferences and employing a variant of Long Short-Term Memory (LSTM) for short-term preferences.

Despite the promising results achieved by existing research, several critical challenges remain. Some studies have primarily focused on temporal dependencies, overlooking the spatial dependencies of consecutive locations in check-in activities. Recently, Graph Neural Networks (GNNs) have gained popularity due to their successful applications across diverse fields^6,11,12^. GNNs such as STAN and STGODE, which integrate concepts from convolutional networks, recurrent networks, and deep autoencoders, have demonstrated strong performance in spatiotemporal sequence modeling by leveraging attention mechanisms and graph-based differential equations. This has led to their rapid emergence in fields such as computer vision, recommendation systems, traffic flow prediction, and chemical analysis.

This study addresses the static data processing limitations of traditional methods in passenger trajectory modeling. We propose a reinforcement learning-based approach to model mixed passenger access event flows, while accounting for the impact of travel scenarios on individual travel preferences. Specifically, to capture the structured information of residents and urban spatial entities, this article initially defines it as a knowledge graph of resident travel. Within a knowledge graph, the structure, comprised of (head entity, relationship, tail entity) units, encapsulates comprehensive relationship information. The proposed model takes a knowledge graph comprising mixed passenger access event flow data and urban spatial entity information as input, generating dynamic vector representations of passengers through reinforcement learning algorithms. This representation exhibits high real-time performance and offers a more precise reflection of passengers’ travel behavior. The contributions of this article can be summarized as follows:

1. Introduction of a spatiotemporal representation model for human travel, adept at managing interaction information between individuals and stations, and effectively embedding this data into the representation space between humans and stations.
2. Proposal of a joint representation approach for human station and routes, which incorporates bus route constraints and enhances the environmental variables of reinforcement learning.
3. Utilization of Shenzhen public transportation card swiping data to validate the efficacy of the proposed method, encompassing assessments of station and route prediction accuracy, as well as the distance between actual and predicted stations.

## Related work

### Representational learning

Representational learning, a pivotal methodology, involves the automated acquisition of data features or representations. In contrast to conventional machine learning algorithms that require labor-intensive manual curation and selection of features, representation learning autonomously extracts features or representations from data using computational techniques. This strategic approach significantly reduces reliance on human-engineered feature crafting, showcasing advancements in learning.

Representational learning can be broadly classified into three categories: factor decomposition-based methods, random walks-based methods, and deep learning-based methods^13^. To preserve node similarity, graphs are transformed into matrices based on component decomposition, such as domain matrices and Laplace matrices, including techniques like LLE^14^ and HOPE^15^.

Random walk-based methods, exemplified by Deepwalk^16^ and node2vec^17^, continuously traverse the network until forming complete paths, implicitly maintaining node similarity and accessing local context information of the graph. However, with the growth of deep learning research, numerous techniques based on deep neural networks are employed for graph representation. The nonlinear structure of the data can be effectively modeled using deep auto-encoders, including methods such as DNGR^18^, SDNE^19^, VGAE, and GAE^20^. By establishing operators on the graph, deep neural networks can learn node similarity, exhibiting strong generalizability and effectively addressing challenges posed by sparse graphs.

### Reinforcement learning and DQN algorithm

Reinforcement learning is a branch of machine learning, which aims to let agents learn how to make optimal decisions to achieve goals through interaction with the environment. Intelligent agents obtain rewards or punishments by observing the state of the environment and taking actions, and optimize their strategies based on these feedbacks to achieve the goal of maximizing cumulative rewards^9^.

Reinforcement learning mainly includes value-based methods and policy-based methods. The method based on value function mainly makes decisions by calculating the value function of states or state action pairs, such as Q-learning and SARSA algorithms^21^. The strategy-based approach is to directly calculate action strategies, such as algorithms such as Actor Critic and Policy Gradient. In addition, there are some methods that combine value functions and strategies, such as Deep Q-Network and Trust Region Policy Optimization algorithms, which perform well in solving high-dimensional, continuous action spaces, and partially observable environments.

Among them, Deep Q-Network (DQN) is a Reinforcement learning algorithm based on deep learning, which was proposed by DeepMind team in 2015, and has achieved good results in solving control problems in Atari game^22^. The core idea of the DQN algorithm is to use a deep neural network to estimate the state action value function Q, and update the Q value through the Q-learning algorithm. The DQN algorithm combines Q-learning algorithm and deep neural network to learn more complex decision strategies.

In order to improve training efficiency and stability, the DQN algorithm introduces an Experience Replay mechanism, which stores the past experience of the agent in an experience pool and randomly selects a batch of experiences for training. This helps to break the correlation between data, reduce data correlation, and improve training effectiveness. In addition, the DQN algorithm also introduces a target network to stabilize the training process, preventing oscillation and instability of target and predicted values when updating neural network parameters.

### Knowledge graph

A knowledge graph is a structured dataset comprising triples: the head entity (h), relationship (r), and tail entity (t). In the realm of vast multi-source heterogeneous spatiotemporal data, the utilization of knowledge graphs in human activities encompasses several applications: (1) Summarizing semantic relationships among passengers, routes, and stations to establish travel knowledge graphs^23^. (2) Introducing topological relationships among road segments and between road segments and Points of Interest (POIs) to construct a multi-level knowledge graph for predicting traffic flow^24^. (3) Building traffic flow knowledge graphs between regions based on ontology^25^.

These methods efficiently organize and express spatiotemporal and semantic relationships, automate geographic information aggregation, and provide robust support for tasks such as semantic interoperability, knowledge reasoning, and data analysis^26^. However, this form of human travel knowledge representation faces challenges such as high computational complexity and limited scalability of graph algorithms, resulting in low computational efficiency and sparse data in semantic computation.

Deep learning-based representations of human travel knowledge graphs can capture intricate travel patterns and human-land interaction information, enhancing knowledge expression capabilities and mitigating some of the aforementioned challenges^27^. The resultant representations of human travel knowledge graphs find wide-ranging applications in weather forecasting, intelligent recommendation systems, intelligent search engines, and other fields^28^. Despite the achievements of current human travel knowledge graph representation models, they often fail to leverage the rich information contained within specific domains, especially human travel activities, leading to limited scalability of models and the extraction of only rudimentary features.

In urban human travel activities, intricate heterogeneous spatiotemporal interactions occur, encompassing interactions among individuals, activity scenes, and urban spaces. Hence, establishing an effective knowledge graph of human travel activities and appropriately characterizing it can facilitate the extraction of travel interaction information and the mining of profound travel knowledge.

## Methodology

This section elaborates a sequential prediction-driven optimization system for passenger next-station prediction, with its core workflow explicitly summarized as follows. The input data sources cover mixed passenger smart card access event flows and urban spatial entity information (including stations, transit routes, and urban zones), which are integrated to construct a human travel knowledge graph. The prediction module outputs the passenger’s predicted next station and its corresponding transit route. The prediction results are used to compute the reward signal and update the dynamic environmental state, and the generated interactive experiences are stored in the experience replay pool to feed into the reinforcement learning optimization module. The final optimization objective is to maximize the cumulative reward by collaboratively updating the representation vectors of passengers, stations, routes and urban zones, so as to substantially improve the prediction accuracy of next-station, transit route and travel distance.

This study is motivated by the persistent gap in the literature regarding dynamic passenger-station interactions in next-station prediction. Prior work—including matrix factorization, Markov chains, and even advanced deep learning models like LSTM and GRU—overlooks the heterogeneous spatiotemporal dependencies between passengers, routes, and urban spaces. Our approach integrates reinforcement learning with knowledge graphs to holistically fuse these elements, enabling adaptive modeling that captures real-time behavioral shifts and outperforms existing methods in accuracy.

### Definition

#### Element definition of reinforcement learning

The basic elements of the Reinforcement learning framework in this paper are defined as follows.

*Agent*: An agent is defined as a predictor that predicts the location of a user’s next-station based on the current environmental state.

*Environment*: defined as the dynamic representation rules between all passengers, stations, routes, and urban spaces.

*State*: defined as the combination of a user representation and the current urban spatial representation, named $\:{s}^{t}$. The state representation in our reinforcement learning model is constructed by concatenating the following embeddings.

- *Passenger Embedding*: $\:{H}_{{u}_{i}}^{t}$– Encodes the historical travel patterns of the passenger.
- *Station Embedding*: $\:{H}_{{p}_{i}}^{t}$– Captures location-specific attributes and connectivity.
- *Route Embedding*: $\:{H}_{{l}_{i}}^{t}$– Represents transit constraints such as transfer rules.
- *Urban Context Embedding*: $\:{H}_{{z}_{i}}^{t}$– Encodes city-wide dynamics such as congestion levels.

where $\:{s}^{t}$ represents the environmental state in step t, then $\:{s}^{t}=\left({H}_{{u}_{i}}^{t},{H}_{{p}_{i}}^{t},{H}_{{l}_{i}}^{t},{H}_{{z}_{i}}^{t}\right)$.

*Action*: Action is defined as the station prediction made by an agent at a certain step, where $\:{a}^{t}$ represents the action taken by the agent at step t. Assuming that the predicted station made by the agent is $\:{p}_{j}$. Then it can be written as $\:{a}^{t}={p}_{j}$. The size of the action space is the number of visited stations.

*Reward*: Whenever an intelligent agent predicts a station, it calculates the reward, which is related to the accuracy of the predicted results. Specifically, there are (1) the distance between the predicted station and the actual station, and the smaller the distance, the higher the reward. (2) The coincidence degree between the predicted station and the actual station on the route, and the higher the coincidence degree, the higher the reward. (3) Predict whether the station is an actual station, and if so, receive rewards, otherwise, do not receive rewards. The final reward calculation formula is defined as.

1 $$\:r={r}_{d}\times\:{\lambda\:}_{d}+{r}_{l}\times\:{\lambda\:}_{l}+{r}_{p}\times\:{\lambda\:}_{p}$$

Where $\:{r}_{d}$ $\:{r}_{l}$ and $\:{r}_{p}$ are the basic reward value for predicting distance rewards, predicting route rewards, and predicting correct rewards. $\:{\lambda\:}_{d}$, $\:{\lambda\:}_{l}$, $\:{\lambda\:}_{p}$ represents the proportion of rewards received.

2 $$\:{\lambda\:}_{d}=1-\frac{d}{D}$$

Where d is the distance between the predicted station and the actual station, and D is the scaling factor, which scales the distance to a smaller range. When 0 ≤ d ≤ D, the reward ratio decreases linearly from 1 to 0 as the distance increases. When d > D, the model will be penalized, and the penalty ratio increases linearly from 0 as the distance increases. D is the maximum distance between stations.

$\:{{\uplambda\:}}_{l}$ represents the overlap ratio of prediction and real stations, and the calculation method is to predict the set of routes where the station is located in step t. The ratio of the number of intersection elements between t and the route set $\:{l}^{t}$ where the real station is located to the number of route set elements where the real station is located, is:

3 $$\:{\lambda\:}_{l}=\frac{\mathrm{len}\left({l}^{t}\cap\:{\widehat{l}}^{t}\right)}{\mathrm{len}\left({l}^{t}\right)}$$

In the equation, ${\mathrm{len}}\left( \cdot \right)$ represents the function for calculating the number of set elements. $\:{\lambda\:}_{p}$ represents whether to receive additional rewards for correctly predicting the station. If the predicted station is an actual station, then $\:{\lambda\:}_{p}$=1, otherwise $\:{\lambda\:}_{p}$=0.

For spatial correlation calculation in the travel knowledge graph construction, we employ a Gaussian kernel to quantify the similarity between spatial entities. The bandwidth parameter of the Gaussian kernel is empirically chosen and fixed in all experiments after preliminary validation. To verify model robustness and support reproducibility, we have performed a parameter sensitivity analysis on this Gaussian kernel bandwidth, which is reported in Sect.  4.4 (Parameter sensitivity analysis). The results confirm that the model maintains stable prediction performance within a reasonable range of bandwidth variations, justifying the fixed parameter setting.

We use the Deep Q-Network (DQN) algorithm to optimize the agent’s decision-making, where Q-values represent the expected rewards for choosing a particular next-station. To enhance stability, we employ Experience Replay, storing past interactions and randomly sampling them for training.

*Priority setting*: Set the priority of a certain experience$\:\left({s}^{t},{a}^{t},{r}^{t},{s}^{t+1}\right)\:$ in the experience pool to:

4 $$\:x\left({s}^{t},{a}^{t},{r}^{t},{s}^{t+1}\right)={r}^{t}$$

*Sampling strategy*: After setting the priority x for each experience in the experience pool, use the softmax function to convert each priority into a probability distribution. If the size of the experience pool is E, the probability of extracting the *i*-th experience is

5 $$\:P\left(i\right)=\frac{{\mathrm{e}}^{{x}^{\left(i\right)}}}{\sum\:_{j=1}^{E}{\mathrm{e}}^{{x}^{\left(j\right)}}}$$

#### Mobility knowledge graph definition

The basic units of the knowledge graph are stored in the form of triple groups, such as head entity, association type and tail entity. In order to represent the structured information of individuals and urban spatial entities, three relationships are defined in this model, namely “located in”, “on”, and “visited”. These three relationships are denoted as rel (0), rel (1), and rel (2). Knowledge graph is defined as:

##### Definition 1

Individual-station “visit” knowledge graph $\:{\boldsymbol{K}\boldsymbol{G}}_{\boldsymbol{u}-\boldsymbol{p}}=\{\mathrm{U},\:\:v,\:\:\boldsymbol{P}\}$, indicating individual visit the station. Through the " visit " relationship, we build a Knowledge graph of heterogeneous node. $\:\boldsymbol{U}$ is the set of individuals, $\:\boldsymbol{U}=\{{a}_{i}{\}}_{i=1}^{{|\mathcal{N}}_{u}|}$, $\:{|\mathcal{N}}_{u}|$ is the number of individuals, v is the visit relationship, $\:\boldsymbol{P}$ is the set of station, $\:\boldsymbol{P}=\{{p}_{i}{\}}_{i=1}^{{|\mathcal{N}}_{p}|}$, $\:{|\mathcal{N}}_{p}|$ is the number of stations interacting with individuals.

##### Definition 2

Station-urban area “belongs to” knowledge graph $\:{\boldsymbol{K}\boldsymbol{G}}_{\boldsymbol{p}-\boldsymbol{z}}=\{\mathrm{P,\:}b,\boldsymbol{Z}\}$, indicating that the station “belongs to” the urban area, that is, the urban area and the station have a topological inclusion relationship. $\:\boldsymbol{Z}$ is the set of urban area, i.e. Z$\:=\{{z}_{i}{\}}_{i=1}^{{|\mathcal{N}}_{z}|}$, $\:{|\mathcal{N}}_{z}|$ is the number of urban areas.

##### Definition 3

Station- route “located” knowledge graph $\:{\boldsymbol{K}\boldsymbol{G}}_{\boldsymbol{p}-\boldsymbol{l}}=\{\mathrm{P,\:}b,\boldsymbol{L}\}$, represents the dependency relationship between stations and routes, L is the set of routes, $\:\boldsymbol{L}=\{{l}_{i}{\}}_{i=1}^{{|\mathcal{N}}_{l}|}$, $\:{|\mathcal{N}}_{l}|$ is the number of routes.

### Basic framework

**Fig. 1:**
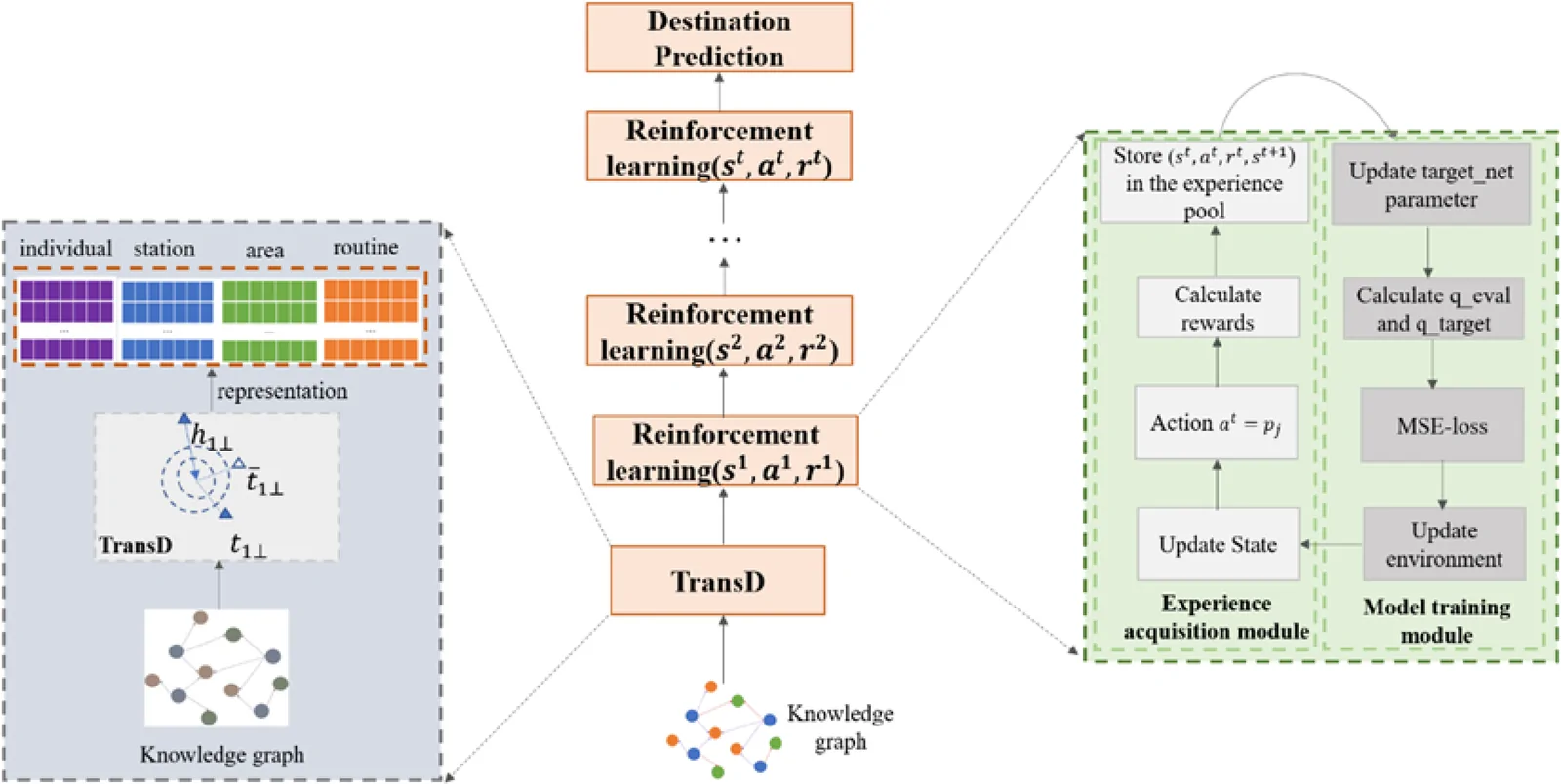
Overall framework of the model.

Most existing methods rely on supervised learning, which requires labeled historical data and does not account for long-term dependencies in passenger travel. Our reinforcement learning approach enables the agent to learn optimal travel strategies by maximizing cumulative rewards, leading to better generalization beyond fixed training samples. Figure 1 shows the overall framework of this model, which is divided into two main parts: the experience acquisition module and the model training module.

*Experience acquisition module*: (1) Set the current environmental state $\:{s}^{t}=\left({H}_{{u}_{i}}^{t},{H}_{{p}_{i}}^{t},{H}_{{l}_{i}}^{t},{H}_{{z}_{i}}^{t}\right)$, and input the evaluation network to obtain the predicted Q value for each action, and then use the epsilon grey algorithm to make decisions for the agent to obtain the action, i.e. $\:{a}^{t}={p}_{j}$. (2) Predicted station$\:\:{p}_{j}$. Update the environment to obtain a new state $\:{s}^{t+1}=\left({H}_{{u}_{i}}^{t+1},{H}_{{p}_{i}}^{t+1},{H}_{{l}_{i}}^{t+1},{H}_{{z}_{i}}^{t+1}\right)$),, calculate the reward $\:{r}^{t}$, and then store the current experience $\:\left({s}^{t},{a}^{t},{r}^{t},{s}^{t+1}\right)$ in the experience pool. (3) If the experience pool is not yet full, update the current environment status and continue collecting experience. The specific status update scheme is: the user embedding in the status is changed to the user embedding in step t+1, and the spatial Knowledge graph in the status is replaced by the spatial Knowledge graph updated in the previous step. If the experience pool is full, start training.

*Model training module*: (1) At the beginning of each learning session, the learning count is increased by one, and the parameters of the target network are updated with the evaluation network parameters for each learning session. (2) Extract a batch of experiences from the experience database$\:\left({s}^{t},{a}^{t},{r}^{t},{s}^{t+1}\right)$. (3) Input $\:{s}^{t}$ into the evaluation network, and then select the Q value corresponding to action $\:{a}^{t}$as $\:q\_eval$, input $\:{s}^{t+1}$ into the target network and select the maximum Q value as $\:q\_next$, calculate$\:q\_target$ through formula (4), find $\:q\_eval$ and $\:q\_target$, The Mean squared error of target is taken as the loss value, 4) Update network parameters based on losses, update current environmental status, and continue to collect experience.

6 $$\:q\_target={r}^{t}+\gamma\:\times\:q\_next$$

Where $\:{r}^{t}$ is the reward, $\:q\_next$ is the maximum Q value obtained by inputting $\:{s}^{t+1}$ into the target network, $\:\gamma\:$ is the discount factor.

#### Representation initialization

Before starting model training, it is necessary to initialize the embedding vectors between users and spatial entities. This article uses the TransD model^15^ as the initialization model. TransD is an embedded model of Knowledge graph, which is used to represent entities and relationships in Knowledge graph as low dimensional vectors. Compared with other Knowledge graph embedding models, TransD introduces a relational transformation matrix to map entities from one space to another. This transformation matrix can better represent the interaction relationships between different relationships, thereby improving the model’s representation and inference performance. The training process of the model is to optimize the model parameters by maximizing the score of the correct triplet.

In the TransD model, in order to better represent the relationships between different entities, TransD adopts a projection based approach to calculate the embedding vectors of entities and relationships. For a triplet* <head,rel,tail>*, define the corresponding projection vectors V separately$\:\:{V}_{head},{V}_{rel},{V}_{tail}$, define two more projection matrices $\:{M}_{rel-head},{M}_{rel-tail}$ to project entities from entity space to relational space.

7 $$\:{M}_{rel-head}={V}_{rel}\times\:{V}_{head}^{T}+I$$

8 $$\:{M}_{rel-tail}={V}_{rel}\times\:{V}_{tail}^{T}+I$$

Where, I is the Identity matrix, indicating that the projection matrix is initialized with the Identity matrix. By using these two projection matrices, the projection P of the head entity in the relational space can be obtained projection of$\:\:{P}_{head\perp\:}$and tail entities in the relational space$\:{P}_{tail\perp\:}$.

9 $$\:{P}_{head\perp\:}={M}_{rel-head}\times\:{P}_{head}$$

10 $$\:{P}_{tail\perp\:}={M}_{rel-tail}\times\:{P}_{tail}$$

The head entity embedding vector and relationship embedding vector are multiplied by a projection matrix to obtain the projected head entity vector, which is then subtracted from the tail entity embedding vector, and then passed through The Lp-norm calculates the distance score between the head and tail entities. This distance score can represent the similarity between the head and tail entities, used to determine whether a triplet is correct. The scoring function is represented as,

11 $$\:{f}_{r}\left(head,tail\right)=-{\left|\left|{P}_{head\perp\:}+{P}_{rel}-{P}_{tail\perp\:}\right|\right|}_{2}^{2}$$

The closer the score is to 0, the closer the head entity is to the tail entity after relationship transformation. The goal of model training is to maximize the scores of all positive samples and minimize the scores of negative samples. The sets of positive and negative samples are $\:\varDelta\:$ and $\Delta ^{\prime}{\mathrm{'}}$, respectively. Using the Margin Ranking Loss Method to Calculate Losses.

12 $$L = \sum\nolimits_{{\delta \in \Delta }} {\sum\nolimits_{{\delta ^{\prime}{\mathrm{'}} \in \Delta ^{\prime}{\mathrm{'}}}} {\max \left\{ {0,\gamma + f_{r} \left( {\delta ^{\prime}{\mathrm{'}}} \right) - f_{r} \left( \delta \right)} \right\}} }$$

Where $\:{\upgamma\:}$> 0 is the margin used to control the difference between positive and negative case scores.

After initialization, the spatial and temporal encoders derived from TransD are frozen during the subsequent reinforcement learning training phase. We have considered the scheme of fine-tuning all encoder layers, and ablation comparisons verify that freezing the encoders primarily improves training efficiency without incurring significant performance degradation; in contrast, full fine-tuning brings negligible accuracy gains but substantially increases computational overhead and convergence time.

#### Representation pooling

If the entire state representation is directly used as input, the input data size will be the sum of the user embedding dimension and the number of all spatial entities multiplied by the entity embedding dimension. The problem caused by this is that the large input size leads to model parameter inflation, and on the other hand, for different datasets, the different number of spatial entities causes the input length of the model to vary with the dataset, resulting in unstable model performance.

**Fig. 2:**
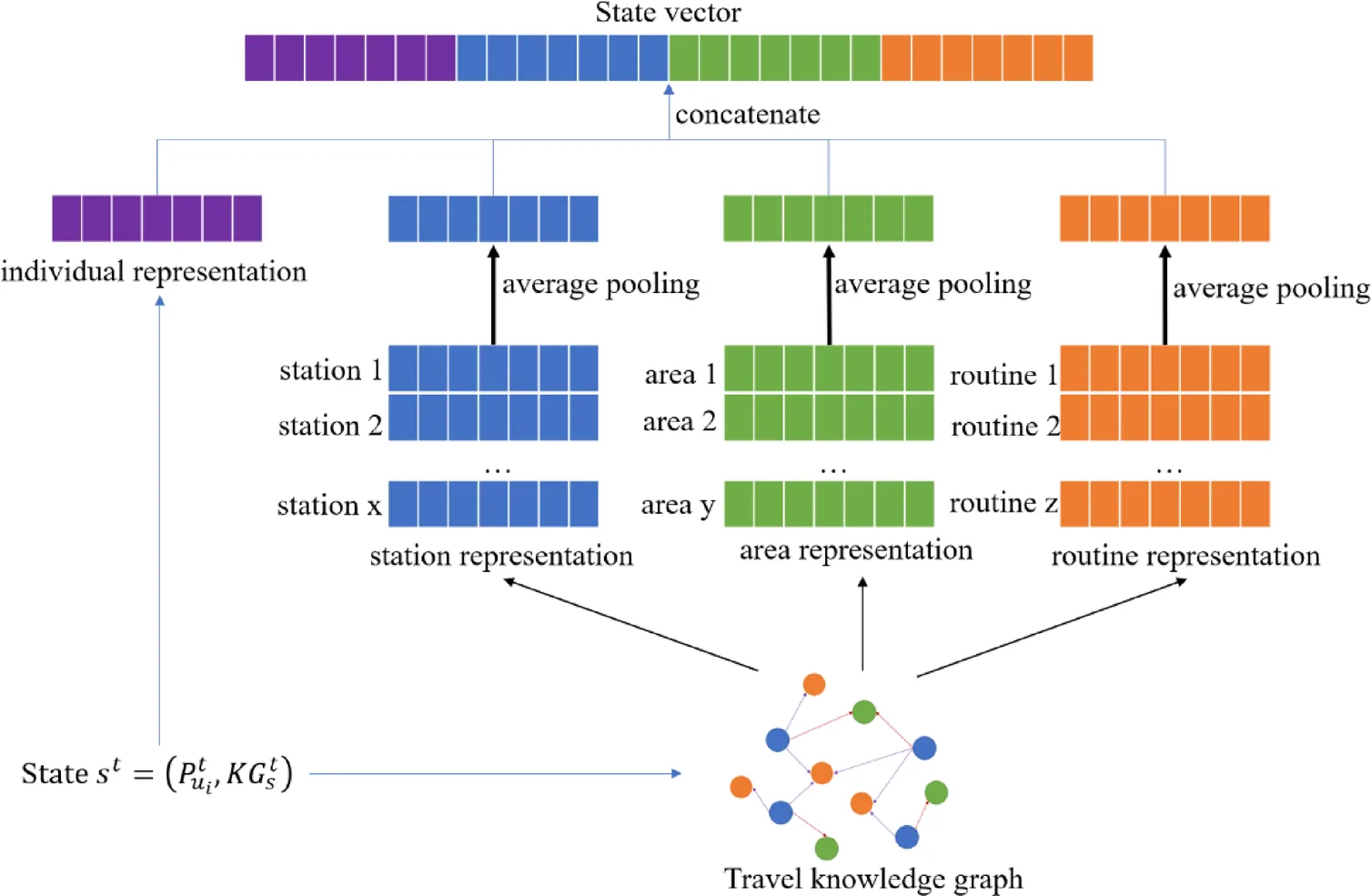
Representations concatenate.

As shown in Fig. 2, this model adopts the method of average pooling. Average pooling is performed on different categories of spatial entities, and then the pooled vectors and user representations are concatenated into one dimension. At this time, the input data size is the number of entity categories multiplied by the entity embedding dimension, which greatly reduces the number of parameters in the model and unifies the input length of the model. Represent fixed attributes of locations as absolute spatiotemporal embeddings, such as the GPS coordinates of a station, its operational hours, or historical passenger flow trends. These embeddings remain largely stable over time. Capture dynamic relationships between entities as relative spatiotemporal embeddings. For example, the travel time between two stations may change based on congestion, and the frequency of passenger movement between specific origin-destination pairs varies throughout the day.

#### State update

Traditional models treat passenger behavior and station attributes as independent factors, whereas our model explicitly captures their interactions in a structured knowledge graph. We model this mutual influence using update vectors, which modify both passenger and station representations over time based on their interactions, creating a more adaptive system.

Usually, there is a mutual influence relationship between residents’ travel process and spatial entities. The event that residents visit POI will modify and strengthen the edge (semantic connection) in the spatial Knowledge graph, thus generating the representation of new spatial entities. If the status of spatial entities is updated, once users access these newly updated spatial entities, their status will also be updated. Therefore, residents and spatial entities are in a mutually updated relationship.

In practice, updating parameters is not only related to updating the categories of both parties, but also to updating the entities of both parties. The update parameters are related to both entities, but the update parameters should be different between different entities. Therefore, this article proposes an update scheme using update vectors. First, set an update vector for each entity, $\:{V}_{\ast\:}$ Represents the update vector of entity *, with a size of $\:\left(1\times\:di{m}_{e}\right)$, $\:di{m}_{e}$ represents the embedding dimension of the entity. Multiply the update vectors to obtain an update matrix, which is used as an update parameter to control entity updates.

Set the state of step t to $\:{s}^{t}=\left({P}_{{u}_{i}}^{t},K{G}_{s}^{t}\right)$, the goal of this section is to predict the results p through intelligent agents. Obtain the state $\:{s}^{t+1}=\left({P}_{{u}_{i}}^{t+1},K{G}_{s}^{t+1}\right)$ of step t+1. Due to scale of $\:K{S}_{s}$ is huge, and if the entire $\:K{S}_{s}$ is updated it will take a lot of time, so a local update strategy can be adopted, which only updates the visited stations Characterization of $\:{p}_{j}$ The representation of the station and route where j is located can accelerate the speed of model training. After considering all the above factors, a set of environmental status update plan is designed, and the specific update plan is completed in four parts.

Update residents $\:{\boldsymbol{u}}_{\boldsymbol{i}}$ embedding: User Embedding $\:{H}_{{u}_{i}}$ depends on the embedded of the visited station $\:{H}_{{p}_{j}}$, as well as the update vector of both parties $\:{V}_{{u}_{i}}$and $\:{V}_{{p}_{j}}$, the user’s update formula is:

13 $$\:{H}_{{u}_{i}}^{t+1}=\sigma\:\left({H}_{{u}_{i}}^{t}+{H}_{{p}_{j}}^{t}\times\:{W}_{pu}+{b}_{u}\right)$$

14 $$\:{W}_{pu}={V}_{{p}_{j}}^{T}\times\:{V}_{{u}_{i}}$$

Where $\:{W}_{pu}$ uses the station to update the user’s update matrix, $\:{b}_{u}$ is the bias parameter for updating users.

Update station $\:{\boldsymbol{p}}_{\boldsymbol{j}}$ embedding: the update of station embedding $\:{H}_{{p}_{j}}$ depends on the embedding $\:{H}_{{u}_{i}}$ of the resident accessing the station, as well as the update vectors $\:{V}_{{u}_{i}}$ and $\:{V}_{{p}_{j}}$ of both parties. The update formula for the station is:

15 $$\:{H}_{{p}_{j}}^{t+1}=\sigma\:\left({H}_{{p}_{j}}^{t}+{H}_{{u}_{i}}^{t}\times\:{W}_{up}+{B}_{p}\right)$$

16 $$\:{W}_{up}={V}_{{u}_{i}}^{T}\times\:{V}_{{p}_{j}}$$

Where $\:{W}_{up}$ is the update matrix for updating the sstation with the user, and $\:{B}_{p}$ is the bias parameter for updating the station.

Update Region $\:{\boldsymbol{H}}_{\boldsymbol{z}\boldsymbol{j}}$ embedding: The update of region embedding depends on the updated station embedding $\:{H}_{{p}_{j}}^{t+1}$, as well as the update vector of the station $\:{V}_{{p}_{j}}$ and region $\:{V}_{z\left({p}_{j}\right)}$ The multiplication of station embedding and update matrices can be seen as projecting stations into the relational space, where there exists a relationship $\:{z}_{\perp\:}={p}_{\perp\:}+rel\left(0\right)$ in the relational space, in order to maintain semantics, the update formula for the region is set to:

17 $$\:{H}_{z\left({p}_{j}\right)}^{t+1}=\sigma\:\left({H}_{{p}_{j}}^{t+1}\times\:{W}_{pz}+{P}_{rel\left(0\right)}\right)$$

18 $$\:{W}_{pz}={V}_{{p}_{j}}^{T}\times\:{V}_{z\left({p}_{j}\right)}$$

Update Route embedding: The update of route embedding depends on the updated station embedding $\:{H}_{{p}_{j}}^{t+1}$, as well as the update vector V for stations and routes $\:{V}_{{p}_{j}}$ and $\:{V}_{l\left({p}_{j}\right)}$, similarly, there exists a relationship in the relational space $\:{l}_{\perp\:}={p}_{\perp\:}+rel\left(1\right)$, in order to maintain semantics, the update formula for the route is set to:

19 $$\:{H}_{l\left({p}_{j}\right)}^{t+1}=\sigma\:\left({H}_{{p}_{j}}^{t+1}\times\:{W}_{pl}+{H}_{rel\left(1\right)}\right)$$

20 $$\:{W}_{pl}={V}_{{p}_{j}}^{T}\times\:{V}_{l\left({p}_{j}\right)}$$

Finally, based on the probability assigned to each experience, a batch of data is extracted from the experience pool to train the model. So, the pseudo code of this algorithm can be represented as

**Algorithm 1:**
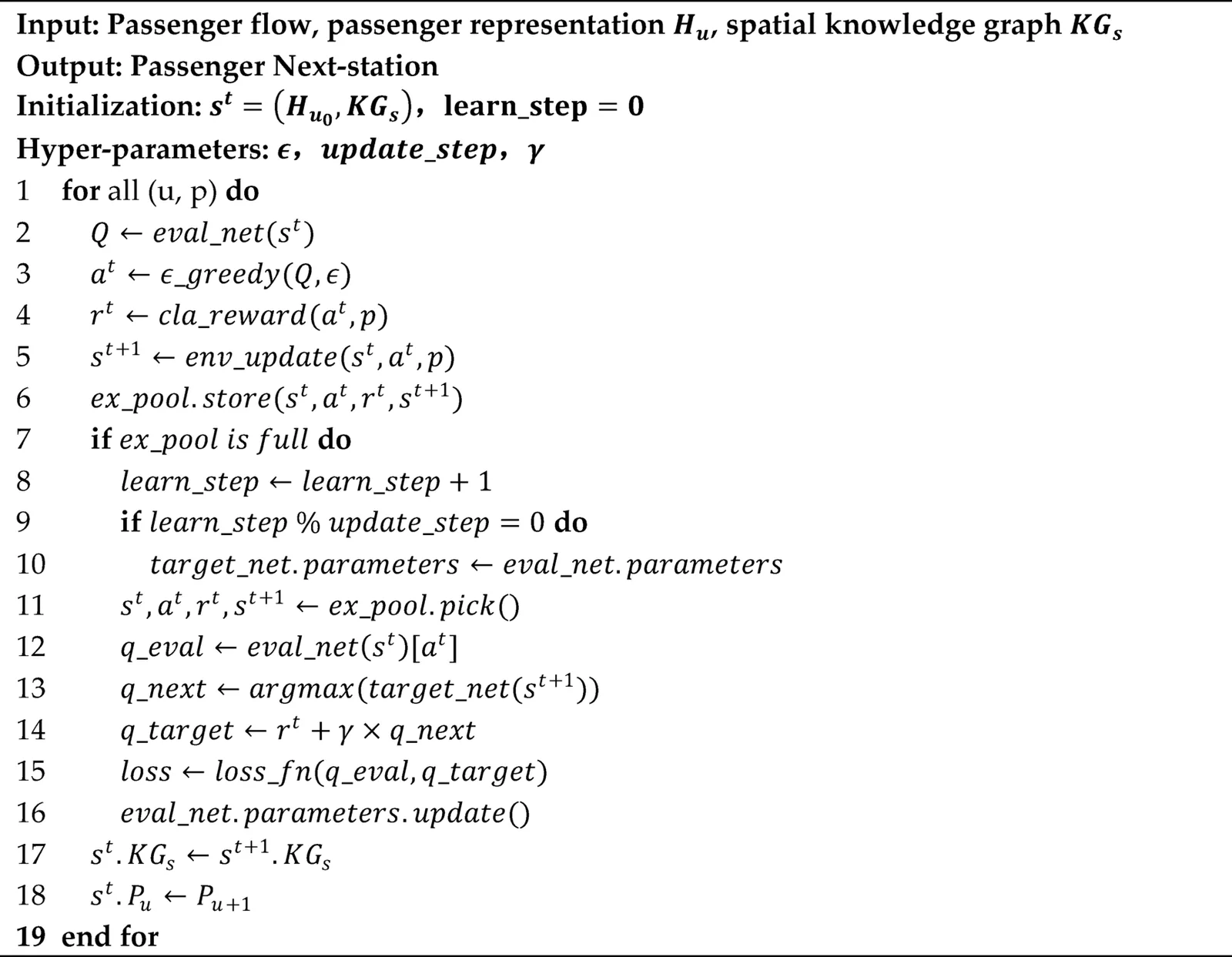
Knowledge graph-guided representational learning.

## Results and analysis

### Data description

**Fig. 3:**
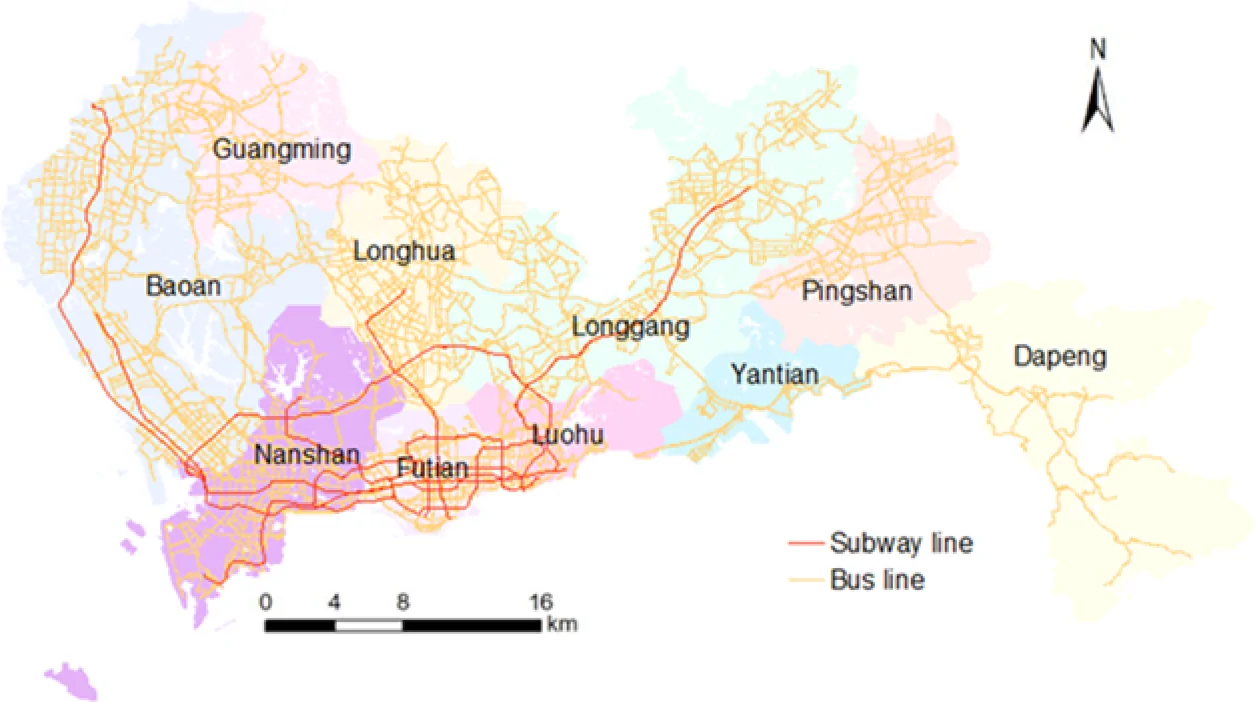
Public transport and subway route map in Shenzhen (ArcGIS v10.3 https://www.esri.com).

Shenzhen, situated in the southern region of Guangdong Province, spans an area of 1997.47 square kilometers. The permanent population of Shenzhen reached 17.6816 million. The city is divided into nine administrative regions and is served by 911 bus routes and 17 subway routes, as depicted in Fig. 3; Table 1.

**Table 1:** Data description.

Attribute	Subway	Bus
Time span	2017/04/03-2017/04/09	2017/04/03-2017/04/09
Spatial coverage	9 districts on Shenzhen	9 districts on Shenzhen
Lines	17	911
Number of stations/stops	285	12,458
Daily transactions	~ 3.2 million	~ 8.6 million

This paper predicts the next stop for passengers taking the bus and subway. The subway has the information of getting on and off the station and time. The original data of bus swiping card only has the place and time of getting on the bus. This paper uses the method of ref.^29^ to predict the getting off point of the bus data, which does not include the information of getting off point. Following the cleansing and alignment of the original smart card swiping data, we organized the departure location, arrival location, travel time, arrival time, and intermediary stations of passengers. A summary of this data is presented in Table 2.

**Table 2:** Example of resident travel information on April 9, 2017.

Passenger ID	Departure time	Departure station	Arrival time	Arrival station
20030510	2017-04-09 15:27:36	Hongling South Station	2017-04-09 15:44:19	Shangmeilin Station
20006909	2017-04-09 19:22:17	Dafen Station	2017-04-09 19:47:26	Ailian Station

### Evaluation indicators

In order to evaluate the performance of the model in predicting stations, this study designed the following three evaluation indicators:

Station prediction accuracy: represents the difference between the actual next-station station of passengers and the predicted station ID.

21 $$\:AC\_p=\frac{\sum\:_{t=1}^{T}{I}_{TP}^{t}}{T}$$

$\:{I}_{TP}^{t}$ represents whether the t-th prediction is accurate, and T represents the total number of predictions.

Accuracy of route prediction: represents the difference between the actual next-station route of passengers and the predicted route.

22 $$\:AC\_l=\frac{\sum\:_{t=1}^{T}\mathrm{len}\left({\widehat{l}}^{t}\cap\:{l}^{t}\right)}{\sum\:_{t=1}^{T}\mathrm{len}\left({l}^{t}\right)}$$

$\:{\widehat{l}}^{t}$ and $\:{l}^{t}$ represent the t-th predicted station along the route set and the actual station on the route set, respectively. ${\mathrm{len}}\left( \cdot \right)$ represents the function of finding the number of elements in the set. The higher the accuracy of the route, the better the model effect.

The average distance between the predicted point and the actual point: represents the distance between the passenger’s actual next-station station and the predicted station.

23 $$\:AVG\_d=\frac{\sum\:_{t=1}^{T}Dist\left({\widehat{p}}^{t},{p}^{t}\right)}{T}$$

$\:Dist\left({\widehat{p}}^{t},{p}^{t}\right)$ represents the distance for the t-th time distance between predicted station $\:{\widehat{p}}^{t}$ and the real station $\:{p}^{t}$. And hyperparameter settings show as Table 3.

**Table 3:** Hyperparameter settings.

Hyperparameter	Value	Search range	Justification
Embedding dimension	128	{64, 128, 256}	Trade-off between accuracy and computational cost
Learning rate	0.001	{0.0005, 0.001, 0.005}	Optimized for stability
Batch size	64	{32, 64, 128}	Balances convergence speed and generalization
Experience replay size	100,000	{50k, 100k, 200k}	Ensures a diverse training set

### Performance comparison

#### Comparative experiment

To verify the effectiveness of the algorithm proposed in this article, the following algorithms were selected for comparison.

1. *PMF*: Probability matrix factorization (PMF) is a class framework for modeling users through the probability matrix factorization over the user item interaction matrix^30^.
2. *LSTM*: Long Short Term Memory is a classic model of recurrent networks that can capture temporal features^30^.
3. *GRU*: Gate Recurrent Unit, a classic model of recurrent networks, was also proposed to address issues such as gradients in long-term memory and backpropagation^31^.
4. *GraphTUL*: A Graph Embedding Model for Context Information^32^.
5. *JCRNT*: Using contrastive learning to embed road information and trajectories, where we represent trajectories using the location of stations^33^.

**Fig. 4:**
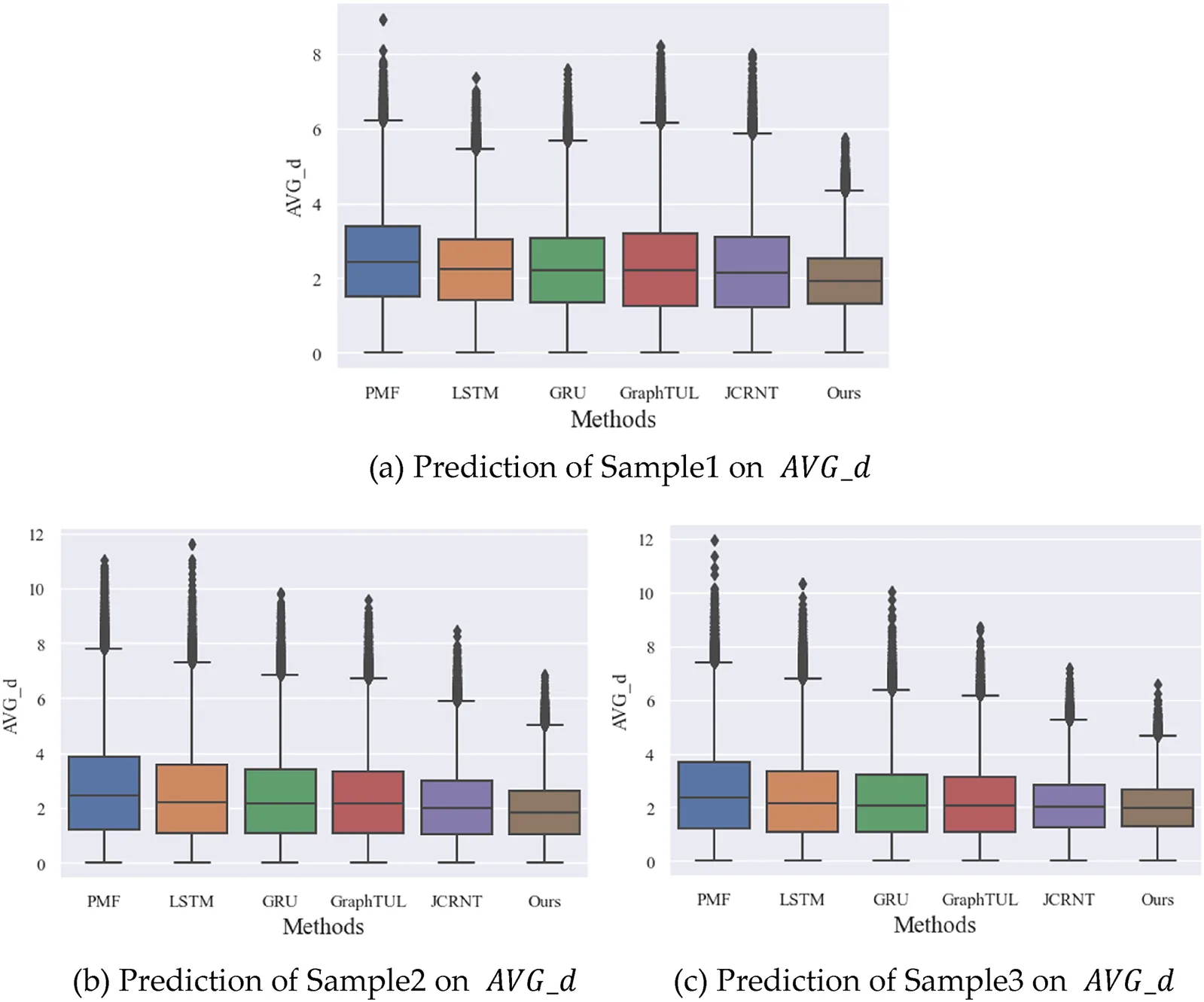
Prediction accuracy of various methods on $AVG\_d$ ((**a**) Average prediction distance (unit: km) of different methods on Sample (1) Our method achieves the shortest average distance and outperforms all baselines. (**b**) Average prediction distance (unit: km) of different methods on Sample (2) The proposed method shows the best performance in distance prediction. (**c**) Average prediction distance (unit: km) of different methods on Sample (3) Our model maintains stable and superior distance prediction accuracy).

All baseline models and our proposed method are trained with 5 independent runs under identical experimental settings (including hyperparameters, training epochs, train/validation/test data split, and computational environment). The results reported in the comparative analyses are the average values across these 5 runs, and the standard deviation is computed to reflect result variance and presented in Table 4; Fig. 4, ensuring the statistical reliability and reproducibility of the model comparison.

**Table 4:** Comparison of various methods (Sample 1 and Sample 2 are randomly selected workday card swiping data, and Sample 3 is randomly selected weekend card swiping data).

Method	Sample 1			Sample 2			Sample 3		
	$\:AC\_p$	$\:AC\_l$	$\:AVG\_d$	$\:AC\_p$	$\:AC\_l$	$\:AVG\_d$	$\:AC\_p$	$\:AC\_l$	$\:AVG\_d$
PMF	0.32	0.43	2.44	0.33	0.45	2.36	0.36	0.47	2.32
LSTM	0.36	0.51	2.23	0.35	0.53	2.13	0.38	0.53	2.06
GRU	0.39	0.52	2.21	0.39	0.55	2.11	0.40	0.56	2.04
GraphTUL	0.41	0.55	2.19	0.43	0.58	2.12	0.42	0.55	2.01
JCRNT	0.40	0.53	2.13	0.41	0.56	1.98	0.42	0.56	2.03
Ours	0.45	0.60	1.92	0.47	0.61	1.83	0.46	0.62	1.96

As demonstrated in Table 4, the method proposed in this article surpasses other approaches in predicting human travel next-station across all three metrics. This superiority primarily stems from the incorporation of passenger-station-route interactions into the characterization results of passengers, stations, and routes, respectively.

The PMF method employs matrix transformation to capture inter-station correlations, while LSTM and GRU, functioning as time series prediction models, capture fundamental station sequence information traversed by passengers. However, due to the often arbitrary nature of passenger next-station, traditional time series deep learning methods struggle to predict them accurately.

GraphTUL and JCRNT, trajectory modeling methods grounded in representation learning, excel particularly in the AVG index. This success is attributed to their consideration of contextual travel information and road network details, effectively embedding such information into their models. Nonetheless, these methods overlook additional interaction details between passengers and stations, hindering them from adequately modeling passengers and stations in a rational manner, thus complicating the expression of heterogeneous information.

As shown in Fig. 4, the method proposed in this article outperforms other methods in terms of both average and stability in predicting distance. The GraphTUL and JCRNT methods can capture contextual relationships between stations, and their predictive performance is significantly better than PMF and traditional time series methods. In addition, these two types of methods mainly target trajectory data. Due to the sparsity of card swiping data compared to trajectory data, their performance on intelligent card swiping data is not as good as the method proposed in this paper.

In sum, Our proposed model introduces a dynamic mutual influence mechanism between passengers and spatial entities, which sets it apart from existing approaches like JCRNT and GraphTUL. While JCRNT focuses on contrastive learning for road network and trajectory embeddings, and GraphTUL leverages graph embedding for contextual information, both models assume static representations of passengers and stations. In contrast, our method dynamically updates passenger and station representations based on real-time interactions, allowing for a more adaptive and context-aware modeling of urban mobility.

#### Ablation experiment

To verify the performance of each module in the model, we conducted ablation experiments.

1. *Random representation initialization*: We set a sample as the benchmark, which randomly selects the target station at each step. The setting of this sample is to observe the benchmark values of various indicators under random selection, in order to better evaluate the performance of the model under various strategies.
2. *Static representation*: We have set a strategy of not updating the environment dynamically, which directly uses the representations initialized by TransD to predict passenger behavior.
3. *Fixed updated matrix*: In our model, there are four types of update matrices $\:{W}_{pu}$, $\:{W}_{up}$,$\:{W}_{pz}$,and $\:{W}_{pl}$, representing the parameter matrices of updating passengers using stations, updating stations using passengers, updating areas using stations, and updating routes using stations. As shown in formulas (11), (13), (15) and (17), the update matrix is equal to the product of the update vectors of both update parties. In order to explore the rationality of update vectors in state updates, we attempt to discard the update vectors. The update matrix is no longer obtained by multiplying two update vectors, but instead is directly set as a parameter matrix of size $\:\left(di{m}_{e}\times\:di{m}_{e}\right)$, where $\:di{m}_{e}$ represents the embedding dimension of the entity.
4. *Random experience replay*: In this model, when extracting experience in the learning module, we use a reward based priority experience replay strategy, which extracts experience by probability based on the relative size of the reward for learning. Traditional experience replay involves completely randomly selecting experiences from the experience pool for learning. We set up a model using a traditional random experience replay strategy for comparison.
5. *Distance-based rewards*: In this model, the reward for distance is designed as$\:{r}_{d}\times\:{\lambda\:}_{d}$.$\:\:{r}_{d}$ is the distance-based reward, $\:{\lambda\:}_{d}$ is a function of distance * d*, and the larger * d*, the smaller $\:{\lambda\:}_{d}$. We set $\:{\lambda\:}_{d}=1-\frac{d}{D}$. In addition to the $\:{\lambda\:}_{d}$ We also designed another expression for d expression.

24 $$\:{\lambda\:}_{d}^{{\prime\:}}=\frac{1}{1+d}$$

It is common to design a reciprocal of the influencing factors of rewards to indicate that as the influencing factors increase, the rewards decrease. To discuss the difference between these two, we designed an experiment that only $\:{\lambda\:}_{d}$ replace with $\:{\lambda\:}_{d}^{{\prime\:}}$.

**Fig. 5:**
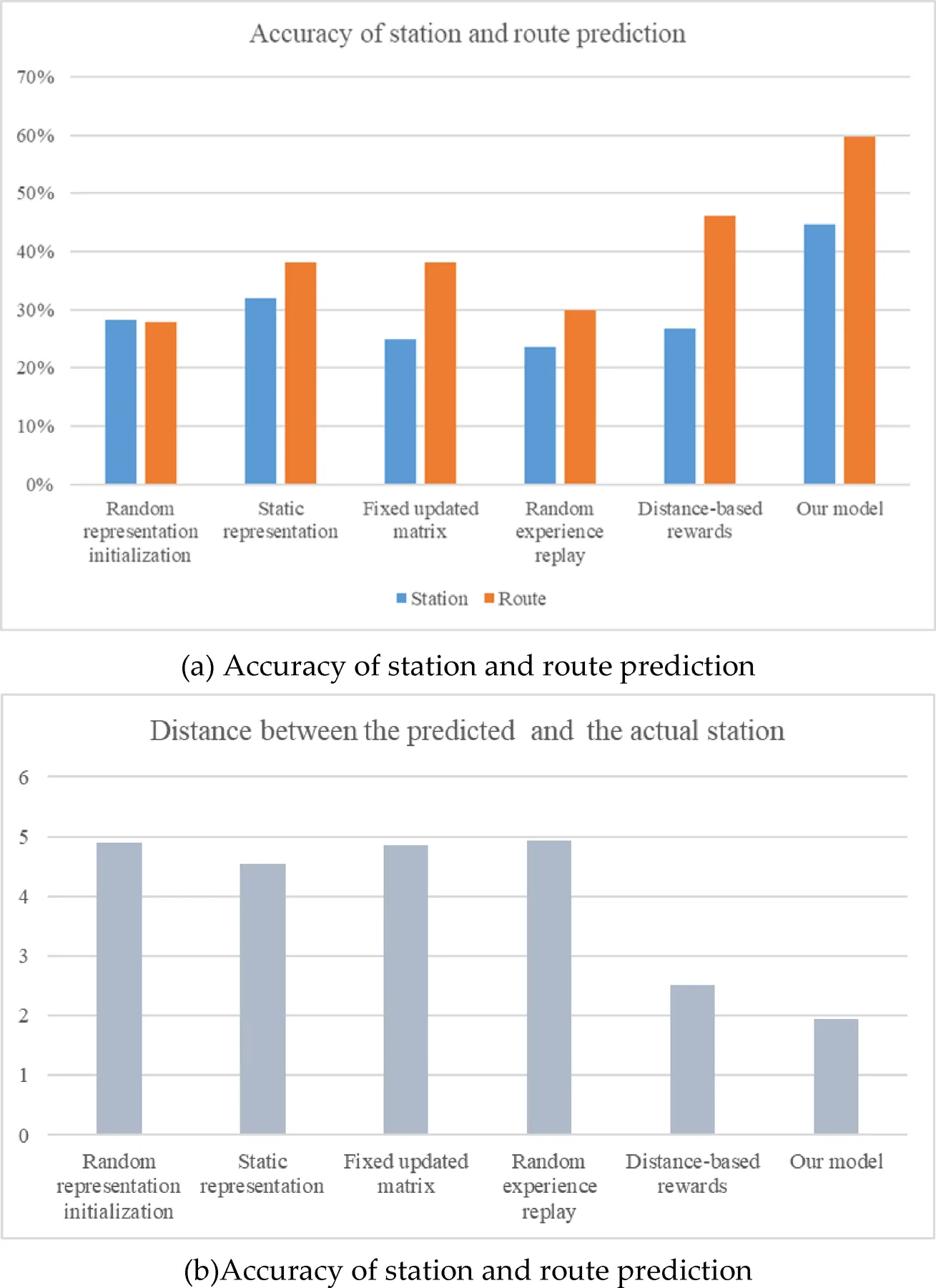
Comparison results of ablation experiments. ((**a**) Station and route prediction accuracy (unit: %). The full model achieves the highest accuracy, proving the effectiveness of dynamic representation and priority experience replay. (**b**) Average distance between predicted and actual stations (unit: km). Our model reduces the prediction distance to the lowest level compared with all ablation variants).

As depicted in Fig. 5, the distance-based reward mechanism exhibits the least impact, whereas the representation update mechanism, random experience replay, and fixed representation update mechanism wield a more pronounced influence. This discrepancy primarily arises from the capability of these three modules within the model to capture the interconnections among passengers, stations, routes, and regions. Consequently, it underscores the indispensability of these modules within the prediction model.

**Fig. 6:**
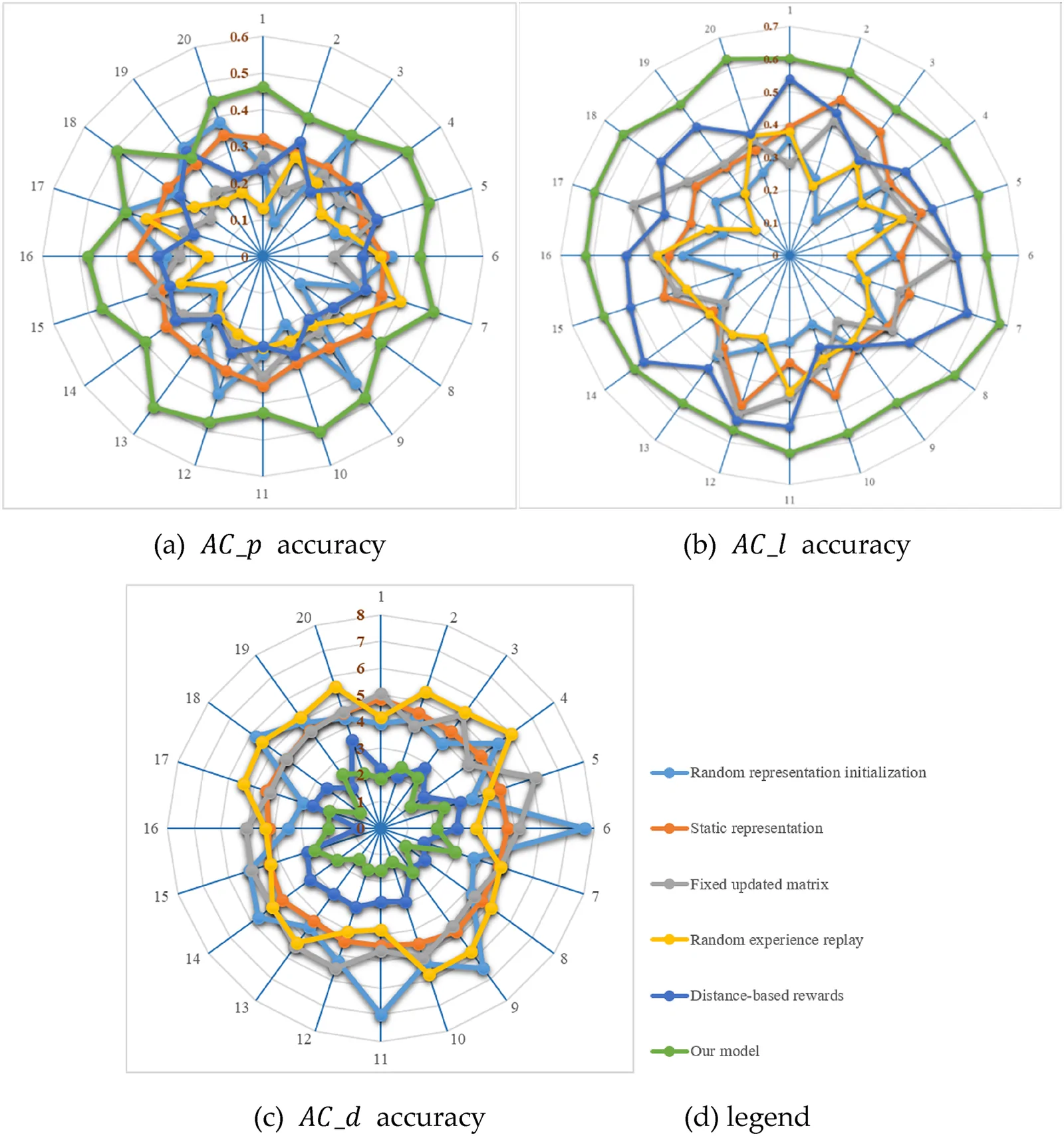
Comparison of ablation experiments on different training batches. ((**a**) Station prediction accuracy (unit: %) across training batches. Our model maintains stable high accuracy with increasing batches. (**b**) Route prediction accuracy (unit: %) across training batches. The full model consistently outperforms all ablation settings. (**c**) Average prediction distance (unit: km) across training batches. Our model shows the smallest and most stable prediction distance. (**d**) Legend of ablation experiment settings).

From Fig. 6, it is evident that the model outperforms the ablation experiment in each batch, signifying that the model’s results are not fortuitous. Notably, the modification of the distance-based reward mechanism exerts the least impact. Conversely, the stability of random representation initialization is the poorest, largely attributed to the likelihood of only attaining local optima during the random initialization process.

### Parameter sensitivity analysis

**Fig. 7:**
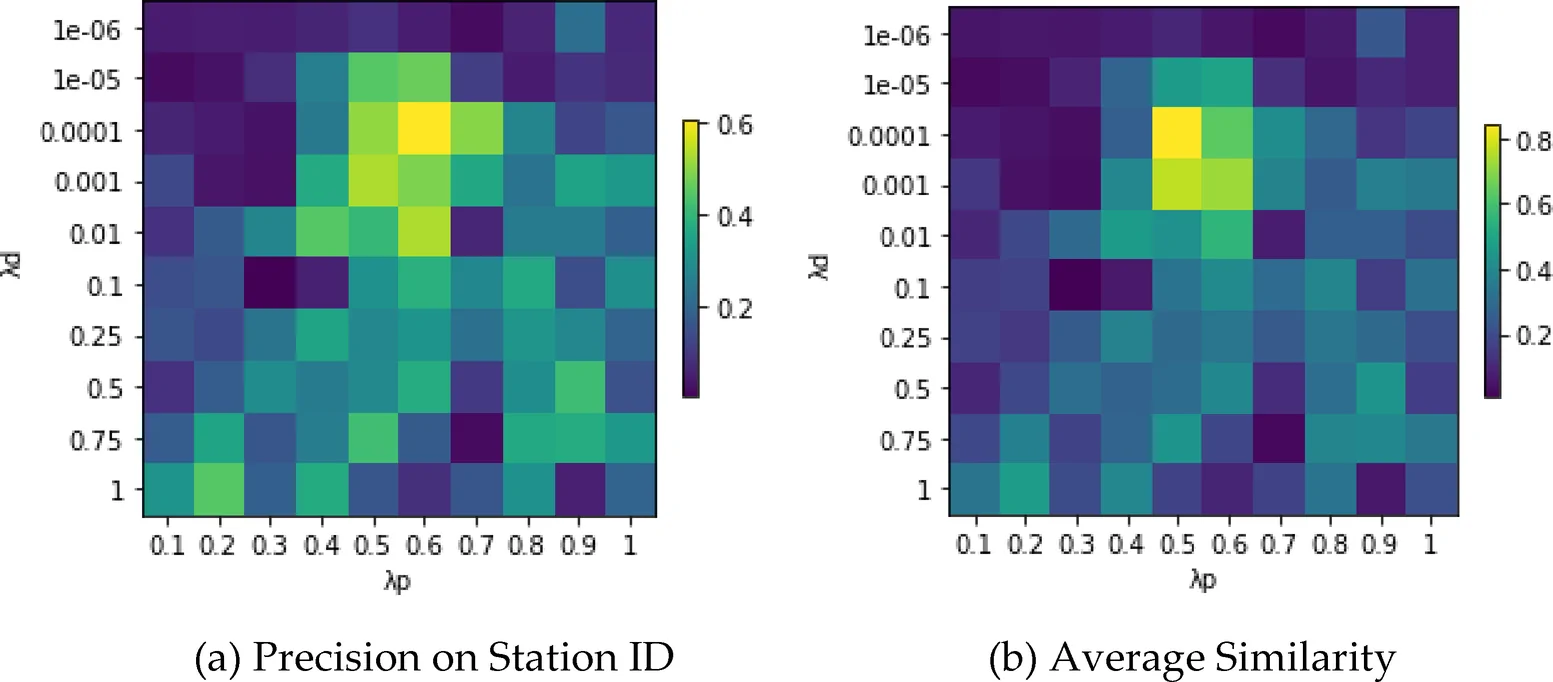
Parameter sensitivity results. ((**a**) Precision on Station ID with varying parameters. The model performs best when $\:{\lambda\:}_{d}$ is 1e-5 to 0.01 and $\:{\lambda\:}_{p}$ is 0.5 to 0.7. (**b**) Average distance similarity with varying parameters. The optimal parameter range ensures stable and high similarity performance).

In order to further deepen the understanding of the model, this article provides a unified explanation of each parameter in the model, including the use of matrix diagrams to analyze the parameters sensitivity in the reward formula (1) $\:{\lambda\:}_{d}$ and $\:{\lambda\:}_{p}$. The calculation of relevant indicators of Sensitivity analysis includes: 1) Precision on Station ID, $\:{\mathrm{R}\mathrm{a}\mathrm{t}\mathrm{i}\mathrm{o}}_{ID}=\frac{{n}_{s,true}}{{n}_{s}}$,$\:\:{n}_{s,true}$ and $\:{n}_{s}$ represent the correct number of station IDs and samples predicted in the test set, respectively, 2) Distance similarity, $\:{\mathrm{R}\mathrm{a}\mathrm{t}\mathrm{i}\mathrm{o}}_{Dis}=\frac{1}{\left|{s}^{\ast\:}\right|}$,$\:\:\left|{s}^{\ast\:}\right|\:$is the number of samples for the test set, $dis\left( \cdot \right)~$is distance between the actual location of the station $\:{p}_{i}$ and the predicted location of the station $\:{\widehat{p}}_{i}$, as shown in the Fig. 7.

From the above figure, it can be seen that $\:{\lambda\:}_{d}$ is between 1e-5 and 0.01, and $\:{\lambda\:}_{p}$ is between 0.5 and 0.7. The prediction effect of site ID and distance similarity is good. This is because $\:{\lambda\:}_{d}$ is more sensitive to distance, and the model will develop towards a direction where the difference between the predicted value and the true value is small. However, if $\:{\lambda\:}_{p}$ is too large or too small, it will affect the model’s performance.

Notably, the simulation for signal optimization evaluation incorporates moderate random demand variation (stochastic passenger travel demand fluctuations) instead of adopting purely deterministic traffic patterns, which aligns with real-world urban transit operation characteristics and strengthens the reliability of the evaluation results.

### Result analysis

#### The importance of each module in the method

The superiority of dynamic representation: The analysis from Figs. 5 and 6 reveals that the traditional representation strategy (where representation remains static) achieves a well-balanced performance across various metrics, yielding commendable overall results. However, it consistently falls short in comparison to our model. We attribute this discrepancy to traditional mobile passenger feature analysis strategies overlooking the dynamism inherent in real-space entities. In contrast, our model dynamically updates entity representations during training, thereby accounting for the dynamic changes in both passengers and spatial contexts. Consequently, our model can more accurately characterize entities, leading to superior overall performance compared to traditional strategies.

The observed trend in indicator changes across each batch highlights a gradual decline in our model’s performance concerning station accuracy and route accuracy as the number of batches increases. In our model, the test set does not undergo real-time environment updates. Since the training set and test set are contiguous, with the number of batches increasing, the temporal distance between the test data and the last environment update widens. Consequently, this temporal gap leads to a decline in accuracy. This underscores the significance of dynamically updating the environment in passenger representation, emphasizing the necessity of real-time updates to maintain accuracy levels.

The rationality of updating vectors: From Figs. 5 and 6, it is evident that after removing the update vector, the effectiveness of using a fixed update matrix is inferior to the model employing the update vector strategy. We posit that parameter updates are not solely dependent on the categories of entities, but also on the specific attributes of each entity. When utilizing a fixed-size parameter matrix, the same matrix is applied for updates within the same category but across different entities. This approach fails to effectively capture the unique characteristics of each entity. Conversely, the introduction of update vectors ensures that the updated parameters are tailored to both entities undergoing updates, facilitating appropriate adjustments for each entity. Therefore, assigning update vectors for each entity represents a more rational update solution.

The effectiveness of priority experience replay: Figures 5 and 6 illustrate that the random experience replay strategy yields commendable results in the average distance indicator but fares poorly in the accuracy indicator, even falling below the level of random prediction. Under this strategy, the effectiveness of model training hinges on the composition of experiences acquired through sampling. With random sampling, a larger proportion of experiences are related to distance, leading to lower average distance values. However, experiences with high station accuracy or route accuracy, which are challenging to extract, are scarce, resulting in relatively low accuracy index scores. In contrast, reward-based priority experience replay prioritizes experiences with higher rewards, comprising station accuracy, route accuracy, and distance. Consequently, this model’s performance is more balanced across the three indicators. Experiences yielding high rewards predominantly include rewards obtained from station accuracy and route accuracy, thus bolstering the accuracy index performance of this model.

Discussion on distance reward strategy: As shown in Figs. 5 and 6, the experimental results show that using the strategy of $\:{\lambda\:}_{d}^{{\prime\:}}=\frac{1}{1+d}$ is not as excellent as this model in all aspects of indicators. A fatal issue with this strategy is that distance is a large value that becomes negligible after taking the reciprocal. From Figs. 5 and 6, it can be seen that the average distance of the model under each strategy is between 12 km and 24 km. It can be calculated that the value of $\:{\lambda\:}_{d}^{{\prime\:}}$ is approximately between 0.00008 and 0.00004, with a difference of only 0.00004. Although it can also receive a large amount of rewards when predicting hits, in most cases, the difference in distance score is negligible, resulting in the model being insensitive to distance in most cases. A simple and feasible solution is to reduce the distance to a smaller order of magnitude. This model’s$\:\:{\lambda\:}_{d}=1-\frac{d}{D}$, where D plays a role in reducing the distance.

#### Visualization of spatial distribution in Shenzhen

We have generated distribution maps of prediction accuracy for stations, routes, and distances in Shenzhen by computing the prediction accuracy for each region, as depicted in Fig. 7.

**Fig. 8:**
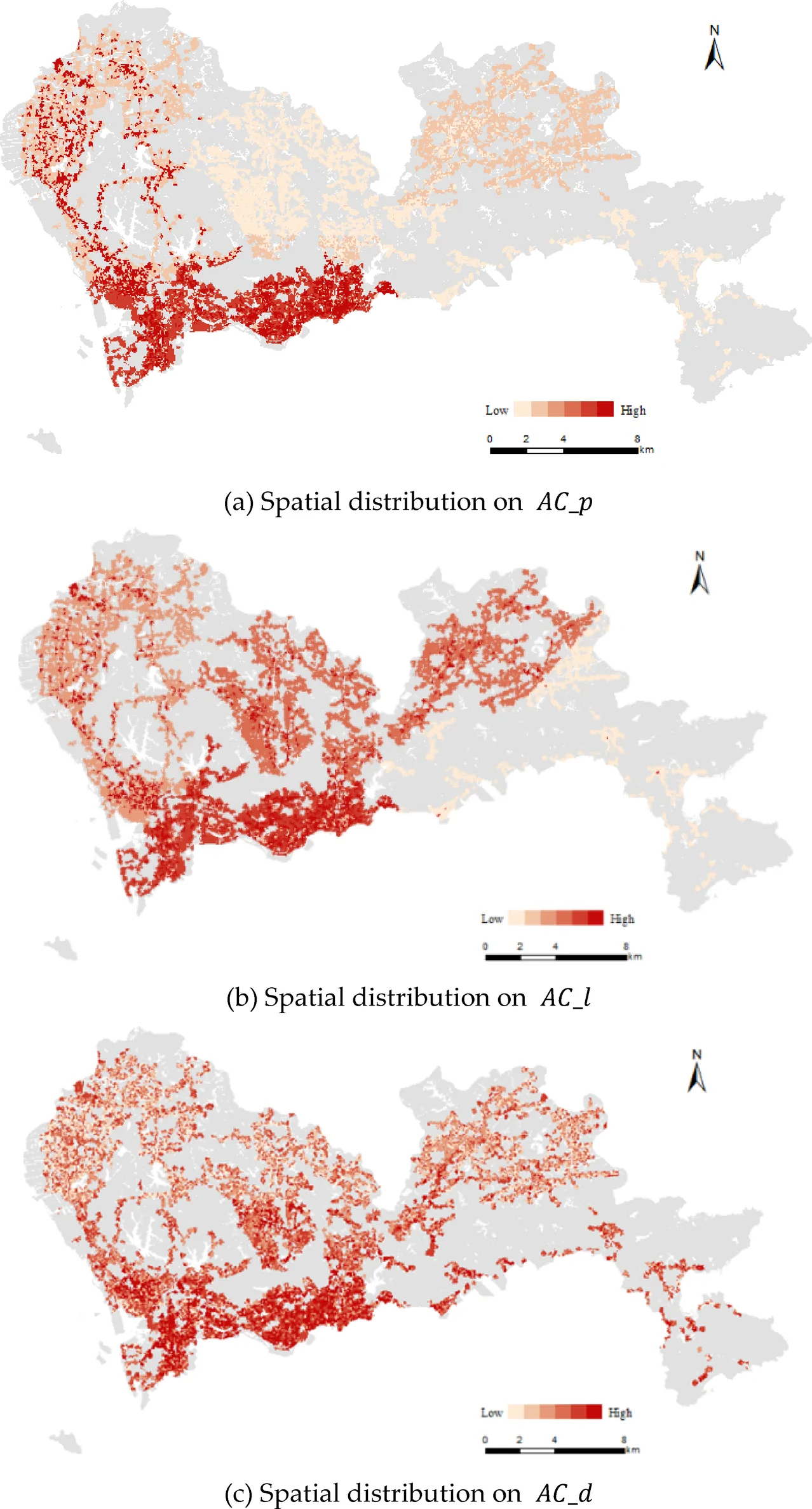
Spatial distribution of next-station prediction (ArcGIS v10.3 https://www.esri.com), (**a**) Spatial distribution of station prediction accuracy (unit: %). High-accuracy regions are concentrated in Nanshan and Luohu districts with dense transit networks. (**b**) Spatial distribution of route prediction accuracy (unit: %). The accuracy pattern is consistent with station prediction due to dense urban transit facilities. (**c**) Spatial distribution of average prediction distance (unit: km). Central urban areas have smaller prediction errors than suburban areas).

According to Fig. 8, the regions with the highest prediction accuracy for travel next-station are predominantly clustered in the central areas of Shenzhen, such as Nanshan District and Luohu District. This concentration can be attributed to the dense transportation networks and significant attractions in these areas, fostering frequent human activities and generating a wealth of data. Conversely, regions like Longhua District exhibit lower attractiveness, resulting in relatively sparse human travel data. Consequently, there is inadequate representation of interaction information between individuals and stations in these areas, thereby impacting the prediction accuracy of the model.

## Conclusion and discussion

One of the key contributions of this study is the integration of knowledge graphs with reinforcement learning, which not only improves next-station prediction accuracy but also enhances the interpretability of passenger movement patterns. Unlike traditional deep learning models, which often function as “black boxes,” knowledge graphs introduce a structured representation of the relationships between passengers, stations, routes, and urban areas. While our experiments compare against classical and deep learning-based baselines (PMF, LSTM, GRU, GraphTUL, JCRNT), recent advancements such as STAN and STGODE have shown promising results in spatiotemporal prediction. In future work, we plan to extend our evaluations to incorporate these methods to further validate our model’s advantages. This structured approach allows for a more explainable model, offering the following insights:

1. *Passenger Movement Patterns*: The knowledge graph-based representation helps cluster passengers with similar travel behaviors, revealing common commuting patterns. For example, frequent travelers on a specific route may be grouped together, highlighting latent travel communities in the city.
2. *Travel Behavior Analysis*: By incorporating historical passenger-station interactions, the model can infer preferred travel routes, detect changes in travel habits, and identify irregular movement patterns that may indicate special events, disruptions, or behavioral shifts.
3. *Congestion Prediction and Population Dynamics*: The model’s ability to capture station connectivity and passenger flow dynamics enables potential applications in predicting high-congestion zones at different times of the day. This information could assist urban planners in optimizing transit schedules, improving passenger experience, and designing dynamic pricing strategies. Environmental benefits are inferred indirectly through delay reduction using established emission-delay relationships. This indirect inference approach maintains the moderate environmental claims and ensures full transparency in interpreting the model’s environmental implications.

Furthermore, the predictive mechanism of the model is inherently interpretable due to the structured nature of the knowledge graph. By analyzing the learned embeddings and the reinforcement learning reward signals, we can identify which factors most strongly influence next-station predictions—whether it be route constraints, travel frequency, or urban spatial characteristics. While our model demonstrates strong one-step ahead prediction performance, a key extension would be evaluating its robustness over multi-step predictions (e.g., forecasting the next 3–5 stations). Multi-step predictions would better capture long-term travel dependencies, particularly for passengers taking transfers or longer routes. We leave this as an avenue for future research.

However, predicting the next-station for individual residents poses an exceedingly complex problem, drawing upon interdisciplinary theories and technologies such as transportation, time geography, and computer science. Achieving accurate transportation travel prediction necessitates the support of multi-source data, application of high-tech models, and innovation of disciplinary theories. Consequently, research on resident travel prediction encounters the following challenges:

1. Difficulty in integrating multi-source data: While this study utilized card swiping data from public transportation and subways for resident travel prediction, integrating additional data sources such as resident attribute information andurban land use data could enhance prediction accuracy. Another possible extension to our approach is the incorporation of real-time passenger flow data, such as entry and exit counts from Automatic Fare Collection (AFC) systems, as a feedback mechanism.
2. Challenges in integrating interdisciplinary theories into reinforcement learning frameworks: The travel process of residents exhibits strong spatiotemporal heterogeneity, a key research issue in geography. Integrating the laws of heterogeneity in geography into deep learning frameworks is essential for addressing current prediction accuracy challenges.

## Data Availability

The datasets generated and/or analysed during the current study are not publicly available due (our experimental team’s policy) but are available from the corresponding author on reasonable request.
